# Wide distribution of carbapenem resistant *Acinetobacter baumannii* in burns patients in Iran

**DOI:** 10.3389/fmicb.2015.01146

**Published:** 2015-10-20

**Authors:** Zahra Farshadzadeh, Farhad B. Hashemi, Sara Rahimi, Babak Pourakbari, Davoud Esmaeili, Mohammad A. Haghighi, Ali Majidpour, Saeed Shojaa, Maryam Rahmani, Samira Gharesi, Masoud Aziemzadeh, Abbas Bahador

**Affiliations:** ^1^Department of Microbiology, School of Medicine, Tehran University of Medical SciencesTehran, Iran; ^2^Department of Microbiology, School of Medicine, Bushehr University of Medical SciencesBushehr, Iran; ^3^Pediatrics Infectious Diseases Research Center, School of Medicine, Tehran University of Medical SciencesTehran, Iran; ^4^Molecular Biology Research Center, Baqiyatallah University of Medical SciencesTehran, Iran; ^5^Anti-microbial Resistance Research Center, Iran University of Medical SciencesTehran, Iran; ^6^Department of Microbiology, Faculty of Medicine, Hormozgan University of Medical SciencesBandar Abbas, Iran

**Keywords:** antimicrobial resistance, MLST, MLVA, international clone, integron

## Abstract

Antimicrobial resistance in carbapenem non-susceptible *Acinetobacter baumannii* (CNSAb) is a major public health concern globally. This study determined the antibiotic resistance and molecular epidemiology of CNSAb isolates from a referral burn center in Tehran, Iran. Sixty-nine CNSAb isolates were tested for susceptibility to antimicrobial agents using the E test methodology. Multiple locus variable number tandem repeat analysis (MLVA), Multilocus sequence typing (MLST) and multiplex PCR were performed. PCR assays tested for ambler classes A, B, and D β-lactamases. Detection of IS*Aba*1, characterization of integrons, and biofilm formation were investigated. Fifty-three (77%) isolates revealed XDR phenotypes. High prevalence of *bla*_OXA-23_-like (88%) and *bla*_PER-1_ (54%) were detected. IS*Aba*1 was detected upstream of *bla*_ADC_, *bla*_OXA-23_-like and *bla*_OXA51_-like genes in, 97, 42, and 26% of isolates, respectively. Thirty-one (45%) isolates were assigned to international clone (IC) variants. MLVA identified 56 distinct types with six clusters and 53 singleton genotypes. Forty previously known MLST sequence types forming 5 clonal complexes were identified. The Class 1 integron (class 1 integrons) gene was identified in 84% of the isolates. The most prevalent (33%) cassette combination was *aac*A4-*cat*B8-*aad*A1. The IC variants were predominant in the *A. baumannii* lineage with the ability to form strong biofilms. The XDR-CNSAb from burned patients in Iran is resistant to various antimicrobials, including tigecycline. This study shows wide genetic diversity in CNSAb. Integrating the new Iranian *A. baumannii* IC variants into the epidemiologic clonal and susceptibility profile databases can help effective global control measures against the XDR-CNSAb pandemic.

## Introduction

*Acinetobacter baumannii* is an important healthcare-associated pathogen that can cause of life threatening infections (Öncül et al., 2014; Girerd-Genessay et al., 2015). *A. baumannii* is the second most common multidrug-resistant cause of nosocomial infection in burn patients in Iran (Alaghehbandan et al., 2012; Azimi et al., 2015). Its infections have become critical challenge to health care systems due to increasing levels of resistance to antimicrobial agents in nosocomial isolates of *A. baumannii* (Cai et al., 2012; Moradi et al., 2015). In developing countries, including Iran, the occurrence of carbapenem non-susceptinle *A. baumannii* (CNSAb) infections is a growing problem in hospitalized burn patients (Mahdian et al., 2015; Zowawi et al., 2015). Inadequate management of the antibiotic therapy of CNSAb infections often leads to the emergence of extensive and pandrug resistant (XDR and PDR) CNSAb strains, which present significant health challenges by prolonging hospitalization, treatment failures, and increased mortality (Liu et al., 2014b). The most important mechanism of carbapenem resistance in *A. baumannii* is the production of carbapenem-hydrolyzing β-lactamases of Ambler classes A, B, and D. In addition to being resistant to all β-lactams available, carbapenemases have a high capacity to spread, since their genes have been commonly found in transferable plasmids containing integrons and insertion sequence (IS) elements (Karah et al., 2012).

Worldwide surveillance has shown that the global population structure of CNSAb isolates is diverse, but a small number of widespread clones, including the international clonal (IC) lineage I–III, also defined based on multilocus sequence typing (MLST) as clonal complexes (CC), may be predominant in healthcare settings (Jeannot et al., 2014). Multilocus variable-number tandem-repeat (VNTR) analysis (MLVA) has been considered as a rapid and cost-effective method for fine-scale typing of *A. baumannii*, enabling inter-laboratory comparison of typing data (Karah et al., 2012). A recent study found the high discriminatory power of MLVA, sufficient to detect diversity among isolates showing identical MLST types (Almeida and Araujo, 2013).

Up-to-date surveillance information regarding genotypic spread, plus local antimicrobial susceptibility patterns of clinical isolates of *A. baumannii*, are necessary for effective drug therapy and control of XDR and PDR *A. baumannii* hospital outbreaks (Fishbain and Peleg, 2010). Considering the points mentioned above, the aim of this study was to determine molecular epidemiology, to identify the dissemination of the most common resistance genes, to investigate the prevalence of integrons and arrangement of integron gene cassettes among CNSAb isolates from a referral burn center in Tehran, Iran.

## Materials and Methods

### Bacterial Isolates

A total of 92 non-repetitive *A. baumannii* strains were collected between January 2012 to May 2013, from the burn wound infections of hospitalized patients in Shahid Motahari hospital, the only referral burn center in Tehran, Iran. Species of these isolates were initially characterized using the API20NE system (bioMérieux, Marcy-l’Etoile, France) and then final identification of isolates were performed by multiplex PCR using *gyrB*-directed primers according to the study of Higgins et al. (2010b).

### Antimicrobial Susceptibility Tests and Biofilm Formation Assay

The Clinical and Laboratory Standards Institute (CLSI) guideline (Clinical and Laboratory Standards Institute [CLSI], 2014) for minimum inhibitory concentrations (MICs) using the E test was used to assess the susceptibility of 92 *A. baumannii* isolates to imipenem (Ezy MIC^TM^ strips, Himedia, India). All isolates with MICs of imipenem >4 mg/L were defined as CNSAb isolates and were recruited for further screening (Clinical and Laboratory Standards Institute [CLSI], 2014).The susceptibility of CNSAb isolates to 17 other antimicrobial agents such as amikacin, ampicillin-sulbactam, cefepime, ceftazidime, ciprofloxacin, colistin, gentamicin, levofloxacin, meropenem, minocycline, piperacillin, piperacillin-tazobactam, rifampicin, tetracycline, tigecycline, tobramicin, and trimethoprim-sulfamethoxazole were also carried out using the E test (Ezy MIC^TM^ strips, Himedia, India). The phenotypic presence of Metallo-β-lactamase (MBL) enzymes was detected by imipenem/imipenem + EDTA strips (Ezy MIC^TM^ strips, Himedia, India). The MIC ratio of MBL-E test of ≥8 mg/L was interpreted as indicative of MBL activity, according to the manufacturer’s instructions. Since there is no breakpoint for tigecycline and rifampicin against *A. baumannii* strains in the CLSI guidelines; therefore, the criteria for interpretation of the MIC values of tigecycline (MIC of ≤1 mg/L defined as susceptible and >2 mg/L as resistant) were determined according to the European committee on antimicrobial susceptibility testing (EUCAST) (European Committee on Antimicrobial Susceptibility Testing, 2014) for members of the *Enterobacteriaceae sp*. and CLSI criteria, using breakpoint values suggested for *Staphylococcus aureus* applied to rifampicin (susceptible defined as ≤1 mg/L and resistant defined as ≥4 mg/L). The MIC_50_ and MIC_90_ of each antibiotic were calculated. *Escherichia coli* ATCC 25922, *Pseudomonas aeruginosa* ATCC 27853, and *E. coli* ATCC 35218 were used as quality control organisms. The phenotype of *A. baumannii* is defined as MDR and XDR according to the International Expert proposal for Interim Standards Guidelines (Magiorakos et al., 2012). The formation assay and quantitative analysis of biofilm were performed according to a previous study (Zhang et al., 2014).

### Molecular Detection of β-lactamases-encoding Genes and Characterization of Integrons

A series of PCR amplifications were done for detection of different Ambler class *bla* gene groups: class A; *bla*_PER-1_, *bla*_V EB-1_, *bla*_CTX-M_, *bla*_KPC_, *bla*_GES_, *bla*_TEM_ and *bla*_SHV -1_ (Poirel et al., 2003; Ellington et al., 2007; Bonnin et al., 2011a,b; Potron et al., 2011; Robledo et al., 2011; Climaco et al., 2013); Class B, *bla*_IMP-1_, *bla*_V IM-2_, *bla*_GIM-1_, *bla*_SPM-1_ *bla*_SIM-1_ and *bla*_NDM-1_ (Chen et al., 2011); Class C, *bla*_ADC_ (Bou and Martinez-Beltran, 2000) and Class D, *bla*_OXA-23_-, *bla*_OXA-40_-, *bla*_OXA-51_-, *bla*_OXA-58_ (Woodford et al., 2006) and *bla*_OXA-143_-like (Higgins et al., 2010a) encoding genes were detected. To determine whether IS*Aba1* was present upstream of *bla*_OXA-23_-like, *bla*_OXA-51_-like (Turton et al., 2006) and *bla*_ADC_ (Heritier et al., 2006) genes, PCR mapping experiments were performed. Detection and characterization of class 1, 2, and 3 integrons, as well as gene cassette mapping and sequencing of class 1 integrons were carried out using previously described PCR assay (Dillon et al., 2005).

### Genetic Diversity and Population Structure

The IC lineage so-called PCR-based group were identified using three-locus dual assay multiplex PCR as described previously (Turton et al., 2007). Isolates pertaining to the novel variant of the PCR-based group, according to the new combination of amplified products obtained from the two separate multiplex PCRs, did not correspond to those previously defined. Moreover, the CNSAb isolates were genotyped using the MLVA-8 scheme method developed by Pourcel et al. (2011). For clustering analysis, the allele strings were entered into the BioNumerics software v.7.0 (Applied Maths, Sint-Martens-Latem, Belgium) as character values. A cut-off value of 90% similarity was applied to define clusters (Nhu et al., 2014). MLST was performed according to the Bartual method described previously (Bartual et al., 2005). Allele sequences, sequence types (STs), primer sequences and other details are available from the MLST website at http://pubmlst.org. The minimum spanning tree (MSTree) algorithm was used to predict the clonal relationship between all CNSAb isolates. MSTree algorithm was constructed with the BioNumerics software v.7.0 (Applied Maths, Sint-Martens-Latem, Belgium) using a categorical coefficient according to a previous report (Ruan et al., 2013).

### Statistical Analysis

Statistical analyses were performed by Student’s *t*-test, Fisher’s exact test and the chi-square test with the SPSS software package (version 16). *P*-values <0.05 in all experiments were considered as significant.

## Results

### Antibiotic Susceptibility Testing and Biofilm Assay

In this study, from the 92 *A. baumannii* isolates, 69 isolates were confirmed as CNSAb. The MIC determination of all CNSAb isolates, exhibited non-susceptibility rates ≥95% to cefepime, ceftazidime, levofloxacin, meropenem, piperacillin, piperacillin-tazobactam, rifampicin and trimethoprim-sulfamethoxazole (**Table 1**). CNSAb isolates showed high susceptibility to colistin (100%), tigecycline (87%) and minocycline (69%) with MIC_50_ values of 0.01, 0.25, and 5 mg/L, respectively and MIC_90_ values of 1, 3, and 10 mg/L, respectively (**Table 1**). Fifty-three (77%) CNSAb isolates revealed XDR phenotypes. In all XDR isolates following colistin with 100% sensitivity, tigecycline (83%) and minocycline (67%) were the most effective antibiotics. Fifty-six (81%) of all CNSAb isolates formed strong biofilm whereas 13 (19%) of these isolates were considered as weak biofilm forming strains. Our study revealed a significant association of strong biofilm formation with antimicrobial resistance (*P* = 0.0001).

**Table 1:** The minimum inhibitory concentration (MIC) distribution of 18 antimicrobial agents for the 69 CNSAb isolates as determined by E test.

Antimicrobial agents^a^	MIC (μg/mL)			Non-susceptible (%)
	Range	MIC_50_	MIC_90_	
CST	0.016 to 2	0.01	1	0
IPM	≤8 *to* ≥ 128	12	32	100
MEM	≤8 *to* ≥ 256	24	48	97
PIP	5 *to* ≥ 240	≥240	≥240	99
TZP	0.01 *to* ≥ 240	120	≥240	96
SAM	2/1 *to* ≥ 256/128	32/16	≥256/128	65
CAZ	4 *to* ≥ 256	128	≥256	96
FEP	4 *to* ≥ 256	128	≥256	97
CIP	0.1 *to* ≥ 240	30	60	95
LVX	0.5 *to* ≥ 240	10	60	95
TET	1 *to* 120	5	30	39
MIN	0.1 to 60	5	10	31
TGC	0.023 to 32	0.25	3	13
TOB	1 *to* ≥ 240	30	120	66
GEN	2 *to* ≥ 240	30	120	93
AMK	8 *to* ≥ 256	64	256	86
SXT	5 *to* ≥ 240	30	60	99
RIF	0.1 to 120	10	30	97

*^a^AMK, amikacin; CAZ, ceftazidime; CIP, ciprofloxacin; CST, colisitin; FEP, cefepime; GEN, gentamicin; IPM, imipenem; LVX, levofloxacin; MEM, meropenem; MIN, minocycline; PIP, piperacillin; RIF, rifampicin; SAM, ampicillin-sulbactam; SXT, trimethoprim- sulfamethoxazole; TET, tetracycline; TGC, tigecycline; TOB, tobramicin; TZP, piperacillin-tazobactam.*

### Detection of Antibiotic Resistance Determinants

**FIGURE 1:**
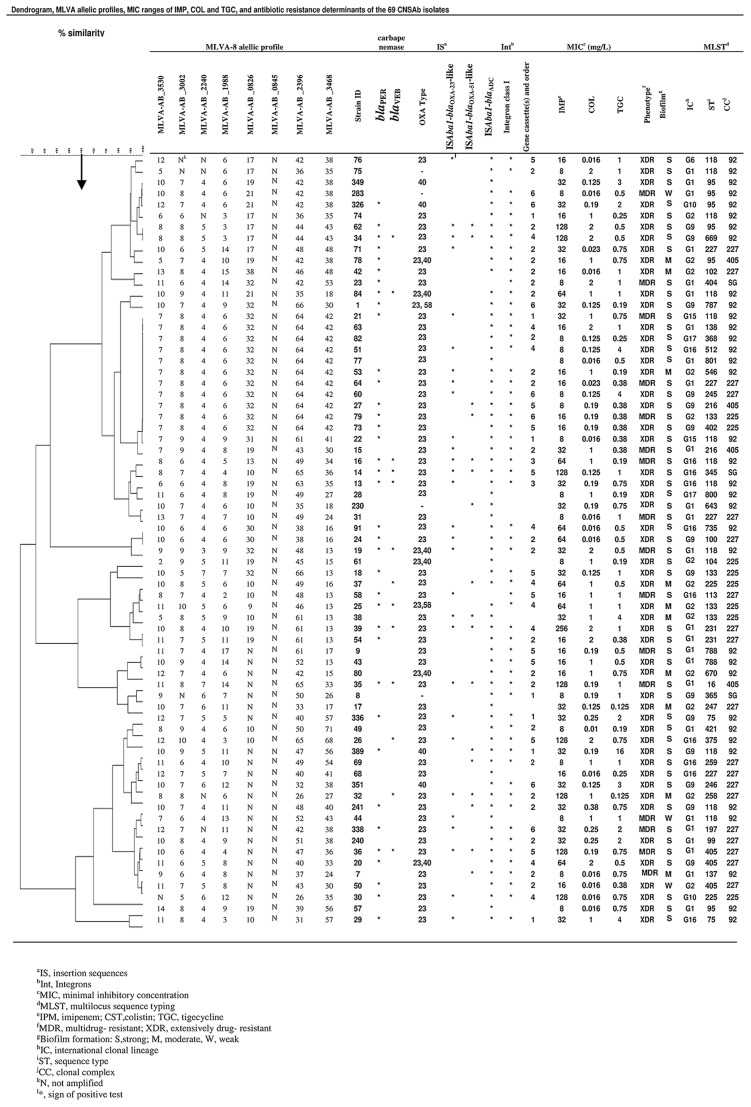
**Dendogram shows the genetic diversity of 69 carbapenem non-susceptible *Acinetobacter baumannii* isolates by MLVA and MLST; carbapenemase encoding genes; MIC ranges of colisitin, imipenem, and tigecycline; resistance phenotype; biofilm formation and international clonal lineage**.

Phenotypic detection of MBL showed that none of the isolates were positive for MBL. The presence of *bla*_OXA51_-like and *bla*_ADC_ were confirmed in all CNSAb isolates. The high prevalence of *bla*_OXA-23_-like (53; 77%) and *bla*_PER-1_ (37; 54%) were seen in CNSAb isolates. Co-existence of *bla*_OXA-23_-like/*bla*_OXA-40_-like and *bla*_OXA-23_-like/*bla*_OXA-58_-like genes were detected in 6 (9%) and 2 (3%) of the isolates, respectively. The PCR results were negative for the other β-lactamase encoding genes including *bla*_OXA-143_-like, *bla* MBL (*bla*_IMP-1_, *bla*_V IM-2_, *bla*_GIM-1_, *bla*_SPM-1_ *bla*_SIM-1_ and *bla*_NDM-_), *bla*_CTX-M_, *bla*_KPC_, *bla*_GES_ *bla*_TEM_ and *bla*_SHV -1._ IS*Aba*1 was detected upstream of *bla*_ADC,_ *bla*_OXA-23_-like, and *bla*_OXA51_-like genes in 67 (97%), 29(42%), and 18 (26%) of CNSAb isolates, respectively (**Figure 1**). A significant correlation was observed between the presence of IS*Aba*1 in upstream of *bla*_OXA-23_-like and increasing MIC of imipenem (MIC ≥ 16 mg/L) (*P* = 0.042). In the presence of IS*Aba*1 in upstream of *bla*_OXA-51_-like and increasing imipenem MICs, there was no significant correlation (*P* = 0.347).

Class 1 integron gene cassettes were identified in 58 (84%) CNSAb isolates while class 2 and 3 integrons were not found. Six different cassette combinations were detected within the class 1 integrons. The most prevalent cassette combination, *aac*A4-*cat*B8-*aad*A1 (2.3 Kb), were detected in 23 (39%) of class 1 integrons. The other cassette combinations including *arr3-aacA4* (1.3 Kb), *aacC1-orfX-orfX’-aadA1* (2.5 Kb), *orfI-aadA1* (1.5 Kb), *dfrVII* (0.7 Kb) *and dfrXII-orfF-aadA2* (1.7 Kb) were detected in 10 (14.5%), 9 (13%), 7 (10%), 7 (10%), and 2 (3%) CNSAb isolates, respectively. The gene cassette arrangement showed that the class 1 integrons harbor genes encoding resistance to aminoglycosides, chloramphenicol, rifampicin and trimethoprim/sulfamethoxazole. Also, the results of this study show that integron-associated β-lactamases encoding genes were not detected in class 1 integrons-harboring CNSAb isolates.

### Genetic Diversity and Population Structure

Three-locus dual assay multiplex PCR showed eight different PCR-based groups (G1, G2, G6, G9, G10, and G15–G17) among the 69 CNSAb isolates. Twenty-five (36%) and 13 (19%) CNSAb isolates belonged to G1 (IC II) and G2 (IC I), respectively. Based on the variations in combination of PCR amplicons of the *bla*_OXA-51_-like, *csuE* and *ompA* genes, 31(45%) of the CNSAb isolates belonged to 6 IC variants PCR-based groups (**Table 2**). G9 (14; 45%) was the most common IC variant (**Table 2**).

**Table 2:** Combinations of amplicons obtained in the dual multiplex PCRs used to describe 31 *A. baumannii* IC variants.

Variant type	PCR group 1			PCR group 2		
	*csuE* 702 bp	*bla*_OXA-51_-like 559 bp	*ompA* 355 bp	*csuE* 580 bp	*ompA* 343 bp	*bla*_OXA-51_-like 162 bp
G 6^a^ (1)	-	-	-	+	-	-
G 9 (14)	-	-	+	+	-	+
G 10 (2)	-	+	-	+	+	-
G 15 (2)	+	-	-	-	-	+
G 16 (10)	-	+	-	+	-	-
G 17 (2)	+	-	+	-	-	+

*^a^G; PCR-based group.*

**FIGURE 2:**
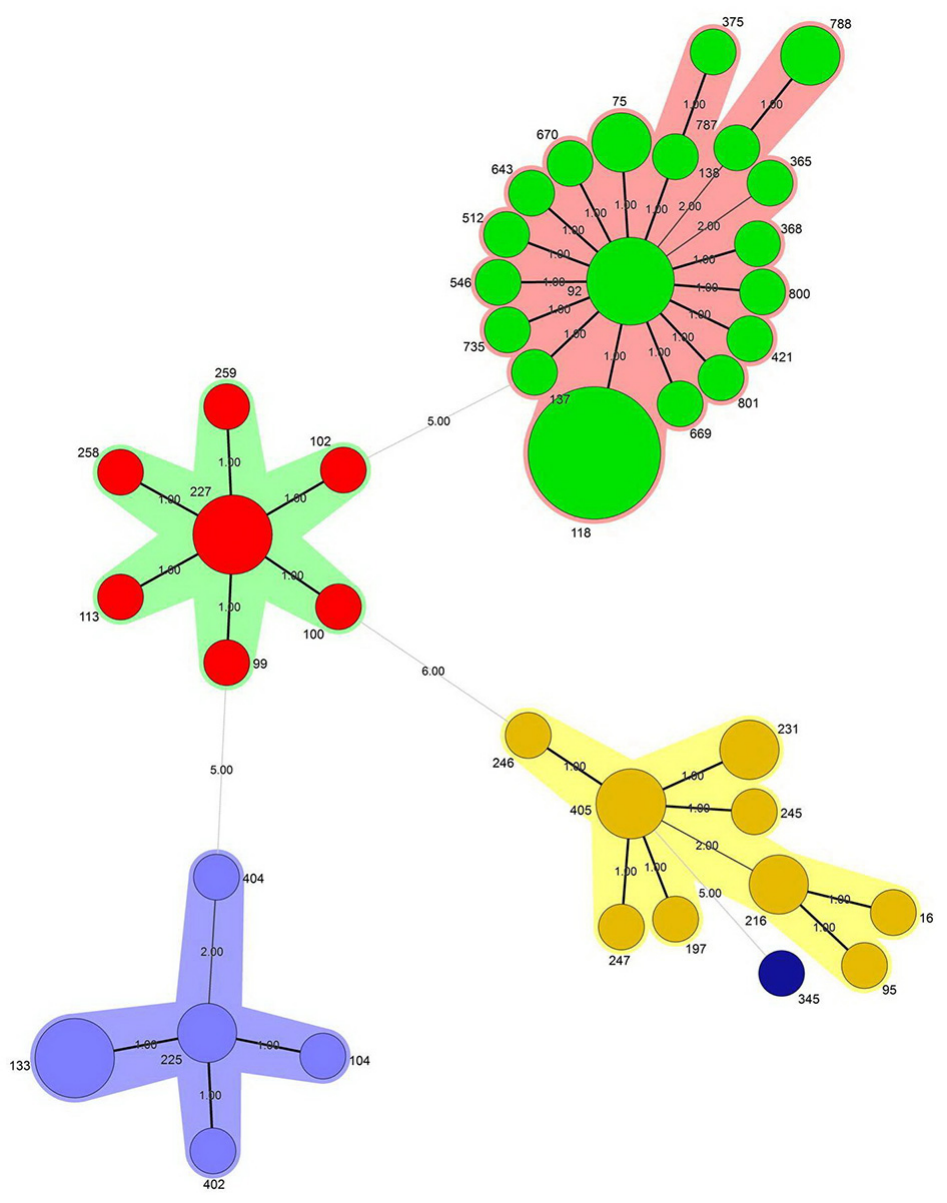
**The minimum spanning tree (MStree) was constructed using a categorical coefficient based on multilocus sequence typing (MLST) data.** Each circle stands for a different sequence type (ST). The color of a circle and the line clustering the ST with the same color represents an identical ST. Size of the circle corresponds to the number of isolates. The width of the line reflects the genetic distance between ST (heavy and thin lines represent a difference in one allele and two or more alleles, respectively).

The MSTree algorithm of the MLST data of this study suggests that 40 previously known STs combined into four CC (**Figure 2**). Thirty-six (52%) and 13 (19%) of all 69 CNSAb isolates belonging to CC-92 and CC-405, respectively. According to the MSTree algorithm, only one singleton ST (ST345) was identified (**Figure 2**). ST118 was the predominant ST, comprising 17.3% of isolates.

By the MLVA typing method, the 69 CNSAb isolates were grouped into 56 distinct MLVA types with 6 clusters and 53 singleton genotypes. Loci MLVA-AB_0845 was absent (N) in all studied isolates. In this study, the VNTR loci MLVA-AB_3002 and MLVA-AB_2240 displayed lower diversity, whereas VNTR loci MLVA-AB_2396 and MLVA-AB_3468 showed higher level of diversity.

Thirty STs corresponded to 30 MLVA types; other STs (118, 95, 75, 227, 405, 231, 788, 225, 227, and 133) included heterogeneous MLVA profiles (**Figure 1**).

In this study, IC variants were classified into 23 STs belonging to CC92, CC405, CC227, CC225 and one singleton (**Figure 1**).

### Relationship between IC Lineages with Antibiotic Resistance Determinants

**Table 3** shows the frequency of antibiotics resistance in two major epidemic lineages and IC variants. Susceptibility to all antimicrobial agents, except tobramicin, in isolates belonged to IC II was higher than the IC I and IC variants (**Table 3**). It is noteworthy that the IC variants had the highest resistance rate to tigecycline (7; 22.5%) with MIC_50_ and MIC_90_ of 0.25 and 3 mg/L, respectively (**Tables 1** and **3**). Among the 13 carbapenem non-susceptible IC I, 12 (92%) were XDR while this rate was 14 (56%) and 27 (87%) in the isolates belonging to the IC variants and IC II, respectively. The resistance to beta lactam antibiotics in IC I and IC variant isolates was higher than that in IC II. Thirty-one (100%) IC variants and 22 (88%) of the IC II population formed strong biofilm but only 3 (23%) of IC I had this characteristic. In the present study, resistance to antimicrobials was comparatively higher among IC variant than IC I and IC II CNSAb isolates (*P* = 0.0012 and *P* = 0.0001, respectively). A slightly higher susceptibility to antimicrobials was observed in IC II than IC variants and IC I (*P* > 0.05). Our analysis revealed a significant correlation (*P* = 0.003) between the presence of *bla*_PER1_and biofilm formation in the CNSAb isolates. The CNSAb isolates belonging to all clones had the *bla*_OXA-23_-like gene. All CNSAb isolates classified in IC I, were positive for the *bla*_OXA-23_-like gene (**Table 4**). According to **Table 4**, the frequency of IS*Aba*1/*bla*_OXA-23_-like and *bla*_PER-1_ genes in a population belonging to IC variants were significantly higher than IC I and IC II (*P* = 0.027 and *P* = 0.003, respectively). **Table 4** shows that the distribution of different types of class 1 integrons is related to major epidemic lineages with predominance of class 1 integrons belonging to type 2.

**Table 3:** Frequency of antimicrobial non-susceptibility in three major epidemic lineages in the 69 CNSAb isolates.

	% Non-susceptibility to CLSI antimicrobial groups^a^																	
IC^b^ (No.)	A								B								O	
	IPM	MEM	CAZ	SAM	AMK	TOB	GEN	CIP	PIP	TZP	FEP	MIN	TET	TGC	LVX	SXT	CST	RIF
IC1 (13)	100	100	100	85	92	62	92	100	100	100	100	48	46	9	100	100	0	100
IC2 (25)	100	92	88	48	84	76	88	88	96	92	96	19	32	5	88	96	0	92
V (31)	100	100	100	61	84	61	100	97	100	97	100	25	39	24	97	100	0	97
Total (69)	100	97	96	65	86	66	93	95	99	96	97	31	39	13	95	99	0	97

*^a^Considerations in the assignment of agents to Groups A, B, and C include clinical efficacy, prevalence of resistance, minimizing emergence of resistance, cost, FDA clinical indications for usage, and current consensus recommendations for first-choice and alternative drugs. **Group A** are considered appropriate for inclusion in a routine, primary testing panel, as well as for routine reporting of results for the specific organism. **Group B** comprises agents that may warrant primary testing. **Group O** (Other) includes agents that have a clinical indication for the organism. *Escherichia coli* ATCC25922 and *Pseudomonas aeruginosa* ATCC27853 were used for quality control of antimicrobial susceptibility testing and included in each run.*

*^b^IC, international clonal lineage; V, IC variants; AMK, amikacin; CAZ, ceftazidime; CIP, ciprofloxacin; CST, colisitin; FEP, cefepime; GEN, gentamicin; IPM, imipenem; LVX, levofloxacin; MEM, meropenem; MIN, minocycline; PIP, piperacillin; RIF, rifampicin; SAM, ampicillin-sulbactam; SXT, trimethoprim- sulfamethoxazole; TET, tetracycline; TGC, tigecycline; TOB, tobramicin; TZP, piperacillin-tazobactam.*

**Table 4:** Frequency of antimicrobials resistance determinants in epidemic clonal lineages of the CNSAb isolates.

IC^a^ (No.)	Ambler classification No. (%)						IS*Aba* upstream beta-lactamase encoding genes No. (%)			Class 1 integron No. (%)	Class 1 integron types^b^ No. (%)					
	Class A		Class D		Co-existence of Class D											
	*bla*_PER_	*bla*_VEB_	*bla*_OXA-23-like_	*bla*_OXA-40-like_	*bla*_OXA-23-40-like_	*bla*_OXA-23-58-like_	IS*Aba*1-*bla*_OXA-23-like_	IS*Aba*1-*bla*_OXA- 51-like_	IS*Aba*1-*bla*_ADC_		1	2	3	4	5	6
IC-I (13)	4 (31)	3 (23)	13 (100)	2 (15)	3 (23)	1 (8)	4 (31)	5 (39)	13 (100)	9 (69)	-	6 (46)	-	2 (15)	1 (8)	-
IC-II (25)	10 (40)	4 (16)	17 (68)	2 (8)	1 (4)	0	7 (28)	7 (28)	24 (96)	19 (76)	1 (4)	8 (32)	-	3 (12)	4 (16)	3 (12)
V (31)	24 (77)	6 (19)	24 (77)	5 (16)	2 (6)	1 (3)	18 (58)	6 (19)	30 (97)	30 (97)	6 (19)	9 (29)	2 (6)	4 (13)	5 (16)	4 (13)
Total (69)	48 (69)	13 (19)	54 (78)	9 (13)	6 (9)	2 (3)	29 (42)	18 (26)	67 (97)	58 (84)	7 (10)	23 (33)	2 (6)	9 (13)	10 (14)	7 (10)

*^a^Abbreviations: IC, international clone; V, variants of IC.*

*^b^Type 1, orfI-aadA1; Type 2, aacA4-catB8-aadA1; Type 3, dfrXII-orfF-aadA2; Type 4, aacC1-orfX-orfX’-aadA1; Type 5, arr3-aacA4; Type 6, dfrVII.*

## Discussion

Nosocomial infections caused by CNSAb are currently among the most difficult to treat. The control of CNSAb infections among hospitalized patients continues to present serious challenges to patient management in developing countries like Iran (Diancourt et al., 2010; Giannouli et al., 2010; Bahador et al., 2013). The main challenge remains choosing the most likely effective antibiotics as determined by *in vitro* testing of antibiotics, based on the local susceptibility patterns (Fishbain and Peleg, 2010). Overall, the findings of this study are consistent with recent studies from Iran that show an alarming trend of CNSAb increase and in MDR *A. baumannii* resistance against a wide spectrum of antimicrobial agents (Moradi et al., 2015). However, in the present study colistin, tigecycline, and tetracyclines exhibited a potent activity against CNSAb isolates. Our data revealed a decrease (10%) of resistance in CNSAb isolates to tigecycline (Bahador et al., 2013), likely due to the implementation of restriction policy on the empirical prescription of tigecycline. Decrease in antibiotic resistance after implementation of restriction policies have been shown in several reports (Altunsoy et al., 2011; Sistanizad et al., 2013). Our finding of positive association in strong biofilm formation and resistance to antimicrobial is consistent with a recent report (Badave and Kulkarni, 2015). This could be due to inadequate penetration of the antimicrobials into the biofilms, and the emergence of persister cells in the biofilms (Penesyan et al., 2015).

The findings of our study on IS*Aba*1 elements upstream of *bla*_OXA_ genes and their role as a promoter to enhance OXA–enzymes expression and MIC of imipenem confirms previous reports (Liu et al., 2014a); however, this finding are in contrast to a recent report by Peymani et al. (2012b) from Iran. Not only did our research find that 42% of CNSAb isolates were the IS*Aba*1-*bla*_OXA-23_-like gene; but also, a significant correlation was observed between the presence of IS*Aba*1 upstream of *bla*_OXA-23_-like gene and increasing imipenem MICs (≥16 mg/L). This dissimilarity might be due to differences in the *A. baumannii* strains available for study. Additionally, our finding of high prevalence of IS*Aba*1 sequences upstream of the *bla*_ADC_ among 97% of CNSAb isolates is consistent with previous report from Iran; and indicate that presence of IS*Aba*1 sequences upstream of *bla*_ADC_ played an important role in increasing resistance to cephalosporins (MIC_50_ = 128 mg/L).

However, IC II is the most common strain in the world (Liu et al., 2014a); and majority of the clinical strains (45%) in this study belong to IC variants. The incidence rate of IC variants observed in this study is similar to previously reported rates of 46% from Sweden and 51% from Iran, and is considerably higher than that in other recent reports, including Iran (3%), Latvia (6%), Romania (15%), Italy (23%) and Norway (28%) (Fishbain and Peleg, 2010; Hojabri et al., 2014; Liu et al., 2014a). With regard to a previous study (Fishbain and Peleg, 2010) and the study of Karah et al. (2012), 14 CNSAb isolates showed a new combination of amplicons and were considered in this article as the PCR-based group (G) 15–17. Three of the 6 PCR-based groups in the present study, namely G6, G9, and G10, have been documented in previous studies (Karah et al., 2012; Bahador et al., 2014). Although there are differences among the IC variant types in this study and previous studies in Iran (Peymani et al., 2012b; Rezaee et al., 2013; Hojabri et al., 2014), the IC variants are highly resistant to antibiotics, which suggests that it is conceivable that the MDR phenotype has substantially contributed to their spread in Iran (Bahador et al., 2014).

The results of this study indicate that, in spite of the high rate of antibiotic resistance determinants in the IC I stains in comparison with the IC variant strains, the IC I strains had lower prevalence. This dissimilarity might be due to the higher frequency of strong biofilm formers in the IC variant strains than in the IC I strains. Several studies proved that, cell adhesiveness and ability to form biofilm were higher in the *bla*_PER-1_ producing isolates (Longo et al., 2014), and this is in agreement with the result of this study. The capability to form biofilm and presence of the *bla*_PER_ gene were significantly higher in IC variants. In this study, the IC II had slightly higher susceptibility to antimicrobial agents than other clonal lineage population. This might be due to low-frequency of carbapenem-hydrolyzing class D β-lactamase (CHDL) genes and IS*Aba*1 elements, as well as low ability of biofilm formation in IC II strain as compared to IC I and IC variant stains.

It has been clearly shown that, the high prevalence of class 1 integrons among MDR *A. baumannii* clinical isolates is responsible for several nosocomial outbreaks globally (Liu et al., 2014b). The resistance rates to all the tested antibiotics, except colistin, tigecycline, minocycline, and tetracycline, of the collected CNSAb isolates were >65%. However, the presence of integron cassette arrays cannot be responsible for all these resistance phenotypes. In Iran, class 1 integrons have been reported previously in >11% of *A. baumannii* isolates (Peymani et al., 2012a; Japoni-Nejad et al., 2013). In the current study, 84% of isolates contain class 1 integrons, but no class 2 and class 3 integrons was detected. This result shows that class 2 and class 3 integrons are not the major resistant determinants in CNSAb isolates in this study, which is consistent with previous studies (Peymani et al., 2012a; Japoni-Nejad et al., 2013).

Only six categories of gene cassettes, including aminoglycoside-modifying enzymes, chloramphenicol-resistant, trimethoprim/sulfamethoxazole-resistant, and rifampicin-resistant were detected in spite of previous studies reporting integrons carrying carbepenemase genes (Japoni-Nejad et al., 2013). This result indicates that acquisitions of carbapenem-hydrolyzing β-lactamase genes plays more important role than the class 1 integrons in carbapenem resistance in CNSAb. The class 1 integrons rate (84%) here defined for a referral burn center in central Iran is higher than the rates found in other geographical regions, including Western Iran (11%) (Salimizand et al., 2014), China (74%) (Wu et al., 2012) and European countries in general (43%) (Castanheira et al., 2014). This result is similar to that of previous studies from the Northwest and center of Iran (Peymani et al., 2012a; Japoni-Nejad et al., 2013). From literature survey, this is the first study to report carriage of class 1 integrons and associated arrays in *A. baumannii* isolates from burn patients in Iran.

Analysis of the integron cassette arrays shows that most class 1 integrons include the globally distributed *aac*A4-*cat*B8-*aad*A1 array (Liu et al., 2014a), and the cassette array *aacC1-orfX-orfX’-aadA1* has been documented in previous studies in Iran (Japoni-Nejad et al., 2013). Moreover, gene cassettes encoding aminoglycoside- resistance are present in the majority of CNSAb isolates in this study, suggesting a close relationship between high-level resistance rate to aminoglycoside compounds such as gentamicin and amikacin and the *A. baumannii* isolates. The different types of gene cassette array in this study and previous studies in Iran (Japoni-Nejad et al., 2013; Salimizand et al., 2014), suggests that a geographical feature plays a major role in MDR isolates formation, and it could be due to different volumes and patterns of antibiotic consumption in distinct areas. In addition, the results of this study indicate that strains indistinguishable from each other by MLST and MLVA typing can have different cassette arrays, indicating that some of these may be transmitted very efficiently. In this study, amikacin resistance recorded in 10% of the CNSAb isolates was not cassette-encoded. Analysis of the integron cassette arrays show that 50% of these isolates harbor an integron without aminoglycoside resistance genes. In the cases where the class 1 integrons could not be amplified, there were no integrons and alteration of the primer binding site or a large size of the Integron are the most likely explanations for the negative result of PCRs.

According to the findings of this research, MLVA could identify closely epidemiologically related isolates clustered by MLST in some cases (**Figure 1**). In this study, isolates with the same ST, such as ST118, have closely related MLVA type (MTs) (**Figure 1**). This result suggests that the evaluation of MLVA in CNSAb isolates shows its great ability for discrimination of genetically closely related isolates.

In agreement with previous studies (Hojabri et al., 2014), the present results substantiated that CC92 represented the recent widely distributed clone in Iran, accounting for 52.2% of CNSAb isolates examined in this study. This most likely indicates that the isolates belonging to CC92 might benefit with respect to acquiring resistance determinants and surviving in the health care environment, when encountering selection by antibiotics such as carbapenems. The international spread of the CC92 isolates has been reported from all around the world (Karah et al., 2012). This study revealed that the 28 isolates belonging to this lineage were shown to be heterogeneous by MLVA (22 MTs), followed by MLST (13 STs). The 69 CNSAb isolates were shown to be heterogeneous by MLVA (56 MTs) and MLST (40 STs). Taken together, genetic diversity and wide dissemination clones among CNSAb isolates is consistent with recent studies from Iran that show high diversity of STs in *A. baumannii* isolates from burned patients (Hojabri et al., 2014), which are the evidence of dynamic population structure and evolution in progress. The referral of patients from other medical centers of Iran to the hospital in Tehran could be among the possible reasons for this diversity and failure to colonize a specific type. Spread of specific clonally related isolates could have been prevented by the success of infection control programs in the teaching hospital in Iran.

## Conclusion

Evidence that XDR-CNSAb from burned patients in Iran is rapidly changing toward growing resistance to various antimicrobials, including tigecycline was presented. Despite the increasing resistance to several first-line antimicrobials, all resistant CNSAb isolates remain sensitive to the antimicrobial colistin, a viable agent in controlling XDR-CNSAb outbreaks, especially in developing countries. This is the first study to perform IC analysis and gene cassette mapping of class integrons of *A. baumannii*, obtained from burned patients in Iran. It can be confirmed that the dominant local IC is an IC variant, with the likelihood that G9 is the predominant member. In the current study, the *aac*A4-*cat*B8-*aad*A1 gene cassette array which confers resistance to aminoglycoside and chloramphenicol was identified as the predominant cassette array of class 1 integrons in CNSAb. This study described the widest distribution of *bla*_OXA-23_-like-horbering IC ST118 (CC92) and ST405 (CC405) were the principal reason for the rapid increase in the carbapenem resistance rate in burned patients in Iran. The findings highlight the importance of an international drug resistance and molecular epidemiology monitoring network of *A. baumannii* isolates to effect global control measures against XDR-CNSAb.

## Conflict of Interest Statement

The authors declare that the research was conducted in the absence of any commercial or financial relationships that could be construed as a potential conflict of interest.
